# Ribosomal Hibernation Factor Links Quorum-Sensing to Acid Resistance in EHEC

**DOI:** 10.3390/microorganisms13081730

**Published:** 2025-07-24

**Authors:** Yang Yang, Xinyi Zhang, Zixin Han, Junpeng Li, Qiaoqiao Fang, Guoqiang Zhu

**Affiliations:** 1 College of Veterinary Medicine, Yangzhou University, Yangzhou 225009, China; 242001110@stu.yzu.edu.cn (Z.H.); mz120231635@stu.yzu.edu.cn (J.L.); mz120241776@stu.yzu.edu.cn (Q.F.); 2 Jiangsu Co-Innovation Center for Important Animal Infectious Diseases and Zoonoses, Joint Laboratory of International Cooperation on Prevention and Control Technology of Important Animal Diseases and Zoonoses of Jiangsu, Yangzhou 225009, China; 3 Agricultural Development Service Center of Liangxi District, Wuxi 214011, China; 19851070082@163.com

**Keywords:** quorum sensing, Ribosomal Hibernation Factor, acid resistance, EHEC, pathogen resilience, SOS

## Abstract

The mechanism by which quorum sensing (QS) enhances stress resistance in enterohemorrhagic *Escherichia coli* (*E. coli*) O157:H7 remains unclear. We employed optimized exogenous QS signal N-acyl-homoserinelactones (AHL) (100 μM 3-oxo-C6-AHL, 2 h) in EHEC O157:H7 strain EDL933, which was validated with endogenous *yenI*-derived AHL, to investigate QS-mediated protection against acid stress. RNA-seq transcriptomics identified key upregulated genes (e.g., *rmf*). Functional validation using isogenic *rmf* knockout mutants generated via λ-Red demonstrated abolished stress resistance and pan-stress vulnerability. Mechanistic studies employing qRT-PCR and stress survival assays established Ribosomal Hibernation Factor (RMF) as a non-redundant executor in a SdiA–RMF–RpoS axis, which activates ribosomal dormancy and SOS response to enhance EHEC survival under diverse stresses. For the first time, we define ribosomal hibernation as the core adaptive strategy linking QS to pathogen resilience, providing crucial mechanistic insights for developing EHEC control measures against foodborne threats.

## 1. Introduction

Enterohemorrhagic *Escherichia coli* (EHEC), particularly serotype O157:H7, poses a severe global threat to public health, largely due to its capacity to cause devastating foodborne illness [1]. Successful host colonization critically depends on the pathogen’s ability to withstand the extreme acidic barrier of the human stomach [2]. Consequently, sophisticated acid resistance (AR) mechanisms are indispensable virulence factors, enabling survival during gastric transit [3]. Although major AR systems such as the glutamate-dependent gad pathway and chaperones like HdeA have been identified, the molecular orchestrators conferring rapid, inducible protection under the dynamic and harsh conditions encountered in vivo remain inadequately defined [4]. Understanding the environmental cues sensed by the pathogens and how they regulate integrated protective responses represents a significant gap in our knowledge of enteric pathogenesis.

Quorum sensing (QS), a cell-density-dependent communication system, regulates collective behaviors essential for bacterial adaptation and virulence across diverse pathogens [5]. In EHEC, which lacks its own AHL synthase, the LuxR homolog SdiA detects exogenous acyl-homoserine lactones (AHLs) produced by other gut microbiota members [6,7,8]. However, a critical unanswered question persists: what downstream effector mechanism(s) link SdiA-mediated QS signal reception directly to the robust activation of AR [5]? Elucidating how EHEC harnesses microbiome-derived QS signals to mount its own life-saving defenses is crucial for deciphering its persistence strategies and identifying potential therapeutic targets. The molecular executor(s) connecting SdiA activation to enhanced AR, and their possible integration with core stress regulators like RpoS [9,10,11,12], remain fundamental unresolved aspects of enteric pathogen adaptation physiology.

While stress transcriptomics in EHEC often implicate ribosomal gene expression changes, the specific functional contribution of ribosomal dynamics—particularly under lethal acidity—is poorly understood [13,14,15]. Research has primarily focused on established AR genes (*gadA*, *hdeA*) [5], yet these systems alone cannot consistently explain the profound resilience observed during severe acid shock or its interplay with concurrent stressors. The significant survival enhancement conferred by exogenous AHLs strongly suggests a functional QS–AR linkage in EHEC [16], but the specific mechanism—how SdiA–AHL sensing translates into cytoprotective physiology—has eluded definition. What is the essential effector pathway downstream of QS?

Growing evidence implicates ribosomal hibernation, an energy-conserving strategy mediated by factors like Ribosome Modulation Factor (RMF) and RaiA, resulting in translationally inactive 100S ribosomes, in stress adaptation beyond simple nutrient deprivation [13,14,15,17,18,19,20]. While stress transcriptomes frequently highlight ribosomal pathways, their definitive role in critical virulence adaptations, particularly acid resistance, is largely speculative. Does QS signaling via SdiA specifically induce ribosomal hibernation as a core survival strategy [5,7,8]? Is this ribosomal reprogramming strictly required for QS-mediated AR, and does it functionally intersect with known AR machinery or the master stress regulator RpoS [9,12]? Moreover, how is this potential pathway regulated by the environmental signal itself—namely, the rapid shift to low pH? Resolving these mechanistic questions is essential to define the hierarchy and integration points within EHEC’s sophisticated adaptive network.

This study aimed to elucidate the molecular mechanism by which QS confers acid resistance in EHEC, with a specific focus on the role of ribosomal hibernation and its master regulator RMF. We sought to determine the following: how SdiA-mediated AHL sensing coordinates RMF-dependent ribosomal remodeling under acidic stress, whether RMF functionally integrates with the global stress regulator RpoS to amplify adaptive responses, and the biological necessity of this pathway for resisting gastric acidity and other host-imposed stresses. Resolving these questions is critical for understanding how pathogens exploit microbiome-derived signals to overcome host defenses—knowledge that could reveal novel targets for disrupting enteric pathogen colonization.

## 2. Materials and Methods

### 2.1. Bacterial Strains and Culture Standardization

The enterohemorrhagic *Escherichia coli* (EHEC) O157:H7 strain EDL933 (ATCC 43895) [21] and its isogenic mutants were preserved at −80 °C in Lysogeny Broth (LB; BD Difco™, Franklin Lakes, NJ, USA) supplemented with 20% (*v*/*v*) glycerol. Prior to experiments, frozen stocks were streaked onto LB agar and incubated aerobically for 12 h at 37 °C. Single colonies were inoculated into 3 mL LB broth and cultured to stationary phase at 37 °C with constant shaking (180 rpm). To ensure phenotypic uniformity, cultures underwent three sequential 1:100 dilutions in fresh LB medium at 12 h intervals. Strains used in this study are listed in Table S1.

### 2.2. Acid Stress Resistance Profiling

Stationary-phase cultures were normalized to OD_600_ = 1.0 in pre-warmed LB (pH 7.4). For acid challenge, 1 mL aliquots were transferred to LB acidified to pH 2.5 or pH 5.5 using sterile HCl (1 N), followed by static incubation at 37 °C. At specified intervals (0, 1, 2, and 3 h), samples were immediately diluted 10-fold in ice-cold phosphate-buffered saline (PBS; 137 mM NaCl, 2.7 mM KCl, 10 mM Na_2_HPO_4_, 1.8 mM KH_2_PO_4_, pH 7.4), subjected to serial dilutions, and plated on LB agar. Viable colonies were enumerated after 12 h of incubation at 37 °C. Survival rates were calculated as CFU at time h (CFU_h_) normalized to t = 0 values (CFU_0_). For quorum sensing (QS) activation, filter-sterilized 3-oxo-C6-acyl-homoserine lactone (AHL; Sigma-Aldrich [#K3007], Merck KGaA, Darmstadt, Germany) was added to final concentrations of 10 μM or 100 μM immediately before acid exposure.

### 2.3. Genetic Strain Construction

Endogenous AHL-producing strain: Plasmid pBR322-*yenI* [22] was electroporated (1.8 kV, 200 Ω, 25 μF; Bio-Rad Gene Pulser^TM^, Bio-Rad Laboratories, Hercules, CA, USA) into chemically competent EDL933 prepared after three ice-cold 10% (*v*/*v*) glycerol washes. Transformants were selected on LB agar containing 100 μg/mL ampicillin (Huaen, Nanjing, China). Functional AHL production was validated by cross-streaking against *Chromobacterium violaceum* CV026 (ATCC 31532), with violacein pigmentation assessed after 24 h incubation at 30 °C [23].

*rmf*-deletion mutant: The λ-Red recombinase system was employed as previously described [23,24]. A 1117 bp chloramphenicol resistance (cat) cassette flanked by 50 bp homology arms targeting the *rmf* locus (amplified with primers P3/P4; sequences in Table S2) was electroporated into EDL933 harboring pKD46 induced with 30 mM L-arabinose for 1 h. Primary recombinants were selected on LB agar containing 30 μg/mL chloramphenicol (Huaen, Nanjing, China). Temperature-sensitive plasmids were eliminated through three successive 8 h cultures at 42 °C. Successful *rmf* deletion was confirmed by PCR amplification of a 168 bp fragment using flanking primers P1/P2 (Table S2), compared to the 253 bp wild-type product (Figure S1).

Genetic complementation: The full-length *rmf* ORF was cloned into the BamHI and SalI restriction sites of pBR322 to generate pBR322-*rmf*. Primers are listed in Table S2. This construct was electroporated into EDL933 Δ*rmf* to create the complemented strain EDL933 Δ*rmf*/*prmf*. For endogenous AHL production in the WT and Δ*rmf* background, pBR322-*yenI* was introduced to generate EDL933 Δ*rmf*/*pyenI* [6]. All constructs were verified by sequencing (Tsingke, Beijing, China).

### 2.4. Transcriptome Sequencing and Analysis

WT EDL933 was exposed to pH 2.5 LB supplemented with or without 100 μM 3-oxo-C6-AHL for 90 min at 37 °C (three biological replicates per condition). Cells were immediately stabilized in RNAprotect Bacterial Reagent (Qiagen, #76506, Hilden, Germany) and total RNA was extracted using TRIzol^TM^ Reagent (Invitrogen^TM^, Thermo Fisher Scientific, Carlsbad, CA, USA) with DNase I treatment (Takara, #2270A, Dalian, China). Ribosomal RNA was depleted using the Ribo-Zero^TM^ Gold rRNA Removal Kit (Illumina, #MRZG12324, San Diego, CA, USA). Strand-specific RNA-seq libraries were prepared with the NEBNext^®^ Ultra^TM^ II Directional RNA Library Prep Kit (NEB, #E7760L, Ipswich, MA, USA) and sequenced (150 bp paired-end) on an Illumina HiSeq 2000 platform (Novogene, Beijing, China), as previously described [25]. HISAT2 (v2.2.1) aligned reads to the EHEC EDL933 reference genome (NCBI RefSeq NC_002655.2). Differential gene expression analysis was performed using DESeq2 (v1.30.1) with thresholds of |log_2_(fold change)| > 1 and a Benjamini–Hochberg adjusted *p*-value (FDR) < 0.01.

### 2.5. Determination of Sublethal H_2_O_2_ Concentration

To establish a physiologically relevant oxidative stress condition, EDL933 WT was cultured in LB broth containing filter-sterilized H_2_O_2_ (Sigma-Aldrich, #H1009, Merck KGaA, Darmstadt, Germany) at final concentrations of 0 (control), 1, 5, or 10 μM. Growth kinetics were monitored by measuring OD_600_ at 2 h intervals for 12 h using a spectrophotometer (BioTek^TM^ Synergy H1, BioTek Instruments, Inc., Winooski, VT, USA).

### 2.6. Multistress Survival Assays

Stress resilience was evaluated under four environmentally relevant conditions. Nutrient deprivation: Stationary-phase cells were washed thrice with PBS and resuspended in 20 mL PBS (pH 7.4) incubated statically at 25 °C [26]. Cold stress: Bacterial suspensions normalized to OD_600_ = 1.0 were held at −20 °C [27]. Thermal stress: Cultures were incubated in a precision-controlled 50 °C water bath [26]. Oxidative stress: Mid-exponential phase cultures (OD_600_ = 0.6 ± 0.05) were exposed to 5 μM H_2_O_2_ in LB broth [28]. Viability was assessed through periodic CFU enumeration as described above in acid stress resistance.

### 2.7. Analysis of the SdiA–RMF–RpoS Regulatory Axis

The pH-dependent signaling circuitry was investigated in WT, Δ*sdiA*, Δ*rmf*, and Δ*rpoS* strains cultured to mid-exponential phase (OD_600_ = 0.6 ± 0.05) in LB at pH 7.0 or pH 2.5, with or without 100 μM 3-oxo-C6-AHL. After 2 h incubation at 37 °C with shaking (180 rpm), cells were harvested for RNA extraction. Gene expression levels of *rmf*, *sdiA*, and *rpoS* were quantified by quantitative reverse transcription PCR (qRT-PCR) as described below [29].

### 2.8. Quantitative PCR and Statistical Analyses

RNA (1 μg) was reverse-transcribed using FastKing gDNA Dispelling RT SuperMix (Tiangen, #KR118-02, Beijing, China). Quantitative PCR was performed in 20 μL reactions containing AceQ^®^ Universal SYBR^®^ qPCR Master Mix (Vazyme, #Q711-02, Nanjing, China) and 0.4 μM gene-specific primers (listed in Table S2). Relative expression was calculated using the 2^−ΔΔCt^ method normalized to *gapA*.

### 2.9. Statistical Analyses

Data represent mean ± standard error of mean (SEM) from at least three independent biological replicates. Statistical significance was determined with an unpaired Student’s *t*-test (two-group comparisons) using GraphPad Prism 9.0 [30]. Significance levels: *p* < 0.05, *p* < 0.01, *p* < 0.001.

## 3. Results

### 3.1. Exogenous and Endogenous AHL Signaling Synergistically Promote Acid Resistance in EHEC

**Figure 1:**
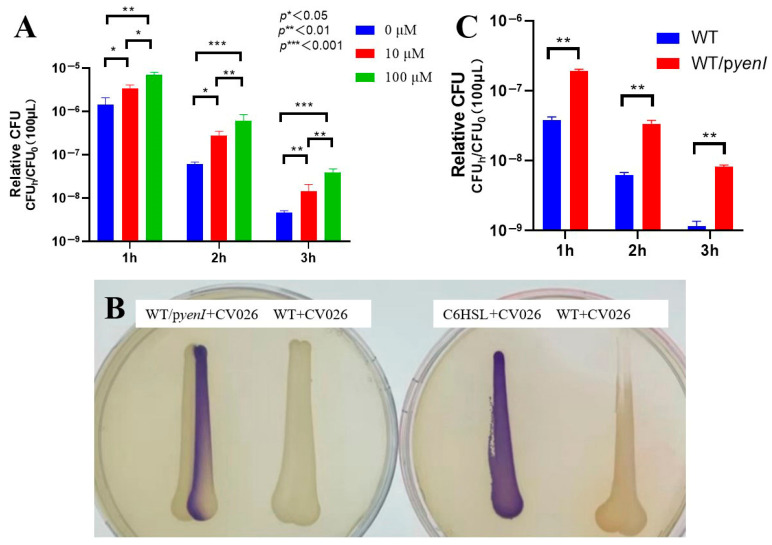
Quorum sensing-dependent enhancement of acid resistance in EHEC. (**A**) Exogenous AHL augments acid survival. Stationary-phase wild-type EDL933 was subjected to pH 2.5 with 0, 10, or 100 μM 3-oxo-C6-AHL for 1–3 h. Survival rates (relative CFU = CFUh/CFU0) were quantified through colony enumeration (n = 3 biological replicates; mean ± SEM; * p < 0.05, ** p < 0.01, *** p < 0.001 versus 0 μM at corresponding time points). (**B**) Endogenous AHL production confirmed by cross-streaking. WT/*pyenI* (left plate) activated violacein biosynthesis in CV026, verifying functional signal synthesis. Controls: C6-HSL-positive control, EDL933 WT-negative control. (**C**) Endogenous AHL signaling promotes acid tolerance. Survival kinetics of WT versus *yenI*-expressing strain (WT/*pyenI*) under pH 2.5 exposure. Relative CFU data demonstrate significant protection by *yenI*-derived AHL (*p* < 0.01 versus WT at matched durations).

We first established the optimal conditions for acyl-homoserine lactone (AHL)-mediated acid resistance in EHEC O157:H7 EDL933. Exogenous 3-oxo-C6-AHL conferred concentration- and time-dependent survival enhancement under lethal acidity (pH 2.5) (Figure 1A): a 10 μM AHL elevated viability of 2.3-fold, 4.5-fold, and 3.2-fold after 1, 2, and 3 h. One hundred μM AHL exerted superior efficacy: 4.9-fold, 9.9-fold (peak at 2 h), and 8.7-fold.

The 2 h timepoint generated maximal protection for both concentrations (4.5- and 9.9-fold). Survival increases with 100 μM AHL significantly exceeded those with 10 μM at 2 h (2.2-fold difference), defining 100 μM AHL + 2 h acid stress as the optimal regimen.

To evaluate endogenous signaling, we engineered the AHL-synthetic strain EDL933 Δ*rmf*/*pyenI*. Cross-streaking assays (Figure 1B) confirmed functional AHL production, as the recombinant strain induced robust violacein pigmentation in biosensor CV026—matching the C6-HSL-positive control. Wild-type EDL933 elicited no response, validating the assay’s specificity.

Endogenous AHL synthesis in WT/*pyenI* significantly enhanced acid resistance (Figure 1C)—1 h: 5.0-fold survival increase; 2 h: 5.5-fold; 3 h: 7.0-fold. Protection escalated temporally (5.0 → 7.0-fold) and surpassed all WT timepoints, demonstrating that *yenI*-driven AHL production confers acid resistance equivalent to 100 μM exogenous supplementation.

### 3.2. Ribosome Hibernation Underpins Quorum Sensing-Mediated Acid Survival in EHEC

**Figure 2:**
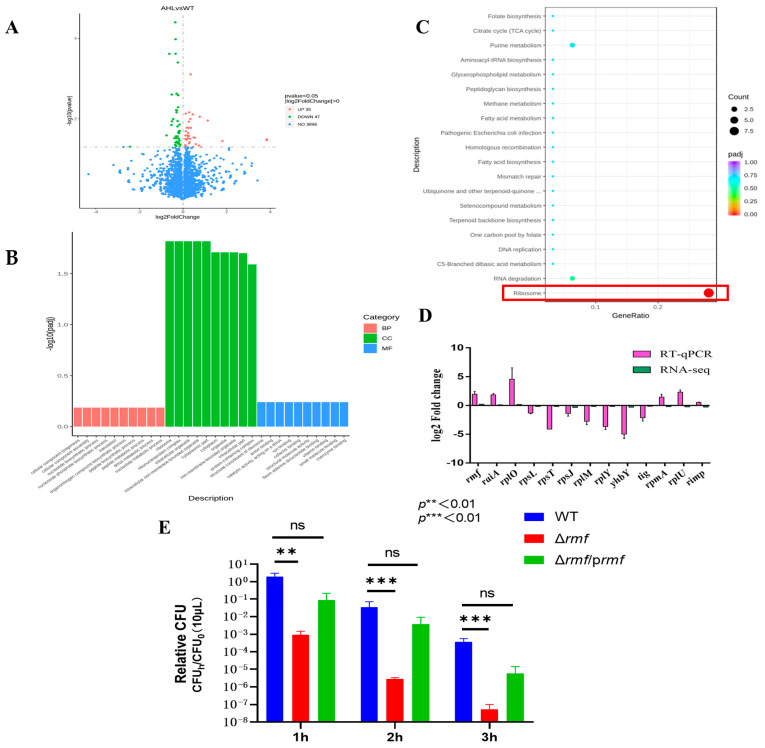
Transcriptomic profiling and functional validation of acid stress response in EHEC. (**A**) Differential expression analysis. Volcano plot identifies significantly altered transcripts between AHL-treated and untreated groups. *X*-axis: log_2_ fold-change; *Y*-axis: −log_10_ (adjusted *p*-value). Red points: up-regulated genes (log_2_FC > 1, FDR < 0.01); green points: down-regulated genes (log_2_FC < −1, FDR < 0.01); blue points: non-significant genes. (**B**) Gene ontology enrichment. Terms are categorized by biological process (red bars), cellular component (green bars), and molecular function (blue bars). Ribosomal pathways show peak enrichment (−log_10_[padj] = 12.1 for cellular components). (**C**) KEGG pathway analysis. Significantly enriched pathways include ribosome assembly (boxed in red; −log_10_[padj] = 10.5) and pathogenic *E. coli* infection. (**D**) Technical validation of RNA-seq data. Log_2_ fold-change comparison between sequencing and RT-qPCR results for selected ribosomal genes (*rmf*, *raiA*, *rpsL*, *rplM*…). Error bars: ±SD. (**E**) Acid resistance impairment in *rmf* mutant. Wild-type (WT), Δ*rmf* deletion mutant, and genetically complemented strain (Δ*rmf*/*prmf*) were challenged at pH 2.5 for 1–3 h. Survival rates (relative CFU = CFU_h_/CFU_0_; mean ± SEM; n = 3) reveal significant attenuation in Δ*rmf* (** p < 0.01, *** p < 0.001 versus WT at corresponding time points).

RNA sequencing of EHEC O157:H7 EDL933 exposed to lethal acidity (pH 2.5) with 100 μM 3-oxo-C6-AHL identified 83 significantly dysregulated transcripts (Figure 2A), comprising 36 upregulated and 47 downregulated genes (|log_2_FC| > 0), while 3896 genes remained unaffected. Some ribosome-related differentially expressed genes are listed in Table 1.

Functional enrichment analyses converged on ribosome pathways. GO terms: Ribosomal structure dominated cellular components (peak −log_10_ (*padj*) = 12.1), translation topped biological processes (8.2), and ribosomal constitution led molecular functions (10.3) (Figure 2B). KEGG pathways: Ribosome assembly (−log_10_ (*padj*) = 10.5) showed >6-fold stronger enrichment than secondary pathways (e.g., RNA degradation: 4.0) (Figure 2C).

Technical validation via RT-qPCR of 13 key ribosomal genes demonstrated (Figure 2D). Coordinated induction of hibernation factors *rmf* (log_2_FC = 2.9 ± 0.2) and *raiA* (2.1 ± 0.3). Suppression of structural ribosomal proteins (*rpsL*: −3.5 ± 0.1; *rplM*: −4.2 ± 0.2). Exceptional consistency between sequencing and qPCR datasets.

Genetic ablation of *rmf* yielded catastrophic acid vulnerability (Figure 2E). After *rmf* deletion, survival plunged 4.4-fold at 1 h, 7.3-fold at 2 h, and 8.0-fold at 3 h vs. the wild-type. A partial restoration of acid tolerance was achieved in Δ*rmf*/*prmf*, with key resistance metrics recovering to 80–89% of wild-type levels.

These data establish ribosome hibernation—orchestrated through *rmf*/*raiA* induction and ribosomal protein suppression—as the principal effector mechanism of quorum sensing-dependent acid resistance.

### 3.3. Ribosomal Hibernation Factor RMF Governs Cross-Stress Adaptation in EHEC

**Figure 3:**
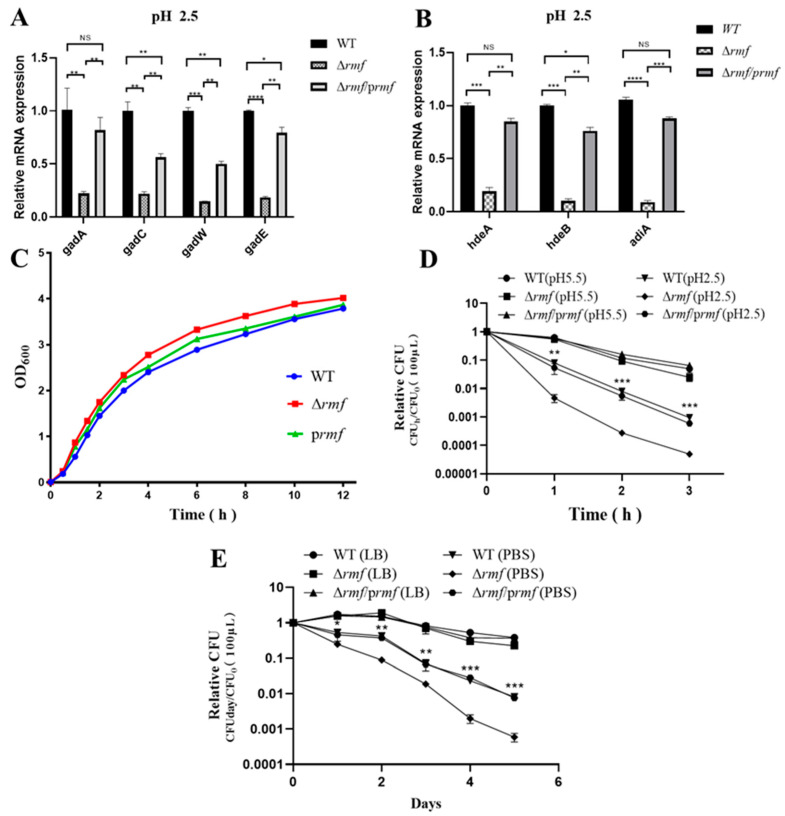
RMF modulates acid-adaptive gene expression and stress survival in EHEC. (**A**) *gad* system dysregulation in Δ*rmf* under lethal acidity. After 2 h at pH 2.5, *gad*A, *gad*C, *gad*W, and *gad*E expression decreased in Δ*rmf* versus WT (* *p* < 0.05, ** *p* < 0.01, *** *p* < 0.001, **** *p* < 0.0001). Genetic complementation (Δ*rmf*/*prmf*) restored baseline levels. (**B**) Impaired chaperone induction in Δ*rmf*. Under identical conditions, *hdeA*, *hdeB*, and *adiA* were downregulated (* *p* < 0.05, ** *p* < 0.01, *** *p* < 0.001, **** *p* < 0.0001). Error bars: ±SD. (**C**) Growth curves. Growth curves show comparable kinetics among WT, Δ*rmf*, and Δ*rmf*/*prmf*. (**D**) Catastrophic acid sensitivity in Δ*rmf*. (**E**) Δ*rmf* starvation vulnerability. In PBS, Δ*rmf* viability dropped versus WT.

Functional dissection established RMF as a master regulator of stress adaptation. The successful deletion of *rmf* in EHEC was verified by agarose gel electrophoresis and sequencing (Figure S1). Targeted control of acid defense: Under pH 2.5 for 2 h, Δ*rmf* showed a significant downregulation of core acid resistance genes: *gadA*, *gadC*, *gadW*, *gadE*, *hdeA*, *hdeB*, and *adiA*. All defects were fully rescued by genetic complementation (Figure 3A,B).

Basal growth restriction: Δ*rmf* exhibited 37% faster maximum growth rate (1.25 h^−1^ vs. WT 0.91 h^−1^), partially reversed in the complemented strain (1.05 h^−1^, 16% slower than Δ*rmf*) (Figure 3C).

Pan-stress survival profiling confirmed RMF’s essential role. In gastric acid stress, Δ*rmf* viability decreased 17-fold after 3 h at pH 2.5, with a time-aggravated collapse (Figure 3D). In nutrient starvation, on day 5 in PBS, Δ*rmf* survival declined 13.7-fold vs. WT, accompanied by 4.8-fold accelerated death kinetics (−0.82 day^−1^ vs. WT −0.17 day^−1^) (Figure 3E). In thermal stress, Δ*rmf* viability was reduced 7.0-fold after 3 h at 50 °C (Figure S2C). In oxidative stress, based on dose-response profiling (Figure S2A), 5 μM H_2_O_2_ was selected for subsequent oxidative stress assays, as it induced ≈50% reduction in stationary-phase biomass (OD_600_ = 2.1 ± 0.2 vs. control 4.0 ± 0.3) without causing rapid lethality (<10% viability loss at 4 h). Under 5 mM H_2_O_2_, Δ*rmf* biomass decreased by 50% at 9 h, partially rescued to 89% of WT levels (Figure S2B). In cryostress, Δ*rmf* survival dropped 6.8-fold after 5 days at −20 °C (Figure S2D).

All stress phenotypes were fully or partially reversed by *rmf* complementation. Crucially, the 13.7-fold viability deficit during starvation (exceeding acid/heat stress impacts) and 4.8-fold accelerated death kinetics position RMF as a primary guardian against energy depletion crises.

### 3.4. RMF Serves as the Essential Downstream Executor of AHL-Mediated Acid Resistance

**Figure 4:**
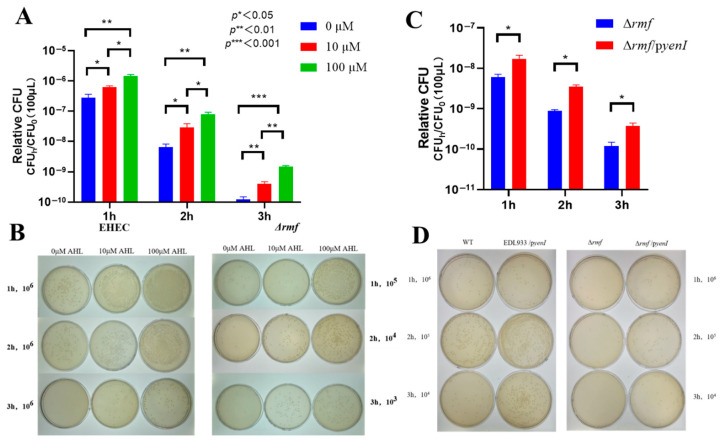
RMF is indispensable for AHL-enhanced acid survival in EHEC. (**A**) Exogenous AHL fails to rescue acid sensitivity in Δ*rmf*. The Δ*rmf* mutant exposed to pH 2.5 for 1–3 h showed minimal protection from 3-oxo-C6-AHL. Data: Relative CFU = CFU_h_/CFU_0_ (mean ± SEM; * *p* < 0.05, ** *p* < 0.01, *** *p* < 0.001 vs. 0 μM at matched time points). (**B**) Endogenous AHL partially restores acid resistance. Complementation with *yenI* (Δ*rmf*/*pyenI*) provided ≤3.9-fold protection after 3 h at pH 2.5 versus 7.0-fold in WT/*pyenI* (* *p* < 0.05 vs. Δ*rmf)*. (**C**) Plate assay demonstrating exogenous AHL efficacy gap. WT maintained confluent growth at 10^6^ dilution after 3 h acid stress with 100 μM AHL, while Δ*rmf* required 1000× higher cell concentrations for detection. (**D**) Endogenous AHL production enhances acid survival. WT/*pyenI* and Δ*rmf*/*pyenI* exhibited denser colonization versus non-AHL-producing counterparts at standardized dilutions (10^4^–10^6^) after 3 h exposure.

Genetic evidence demonstrates that RMF is indispensable for AHL-conferred acid protection in EHEC. Blunted AHL efficacy was displayed in Δ*rmf* (Figure 4A). One hundred μM AHL induced maximal 2.5-fold survival increase (3 h) in Δ*rmf*, representing only 28% of the WT response (8.7-fold). Peak protection delayed to 3 h (vs. 2 h in WT), with 2 h efficacy reduced to 22% (2.2-fold vs. WT 9.9-fold). In colony enumeration assays (Figure 4B), Δ*rmf* required 1000× higher cell concentrations for detection after 3 h of acid stress. One hundred μM AHL generated <30 CFU in Δ*rmf* vs. confluent growth in WT at an identical dilution (10^6^). Endogenous AHL showed limited rescue ability (Figure 4C). *yenI*-expressed AHL in Δ*rmf* background provided ≤3.9-fold protection (vs. 7.0-fold in WT/*pyenI*). Viability reached 0.1% of WT/*pyenI* levels at 3 h. At matched dilutions, Δ*rmf*/*pyenI* exhibited sparse colonies vs. confluent growth in WT/*pyenI* (Figure 4D).

Collectively, *rmf* deletion abolished 72–99.9% of AHL-mediated acid resistance, establishing RMF as the non-redundant effector of quorum sensing signaling.

### 3.5. Acid-Triggered SdiA–RMF–RpoS Signaling Axis Governs Ribosomal Stress Adaptation

**Figure 5:**
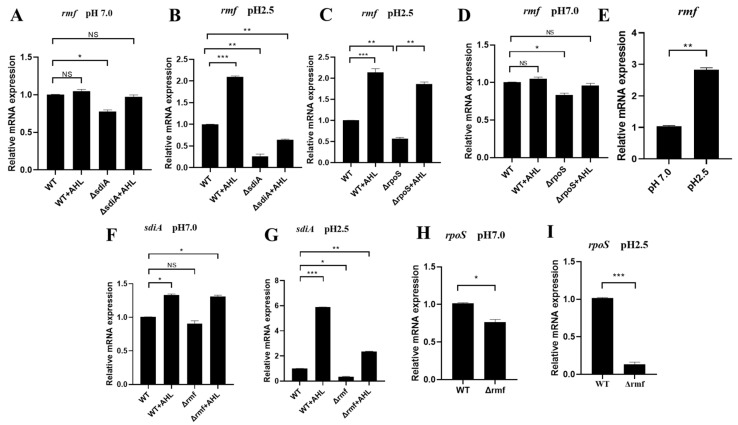
pH-dependent transcriptional circuitry of the SdiA–RMF–RpoS regulatory axis. (**A**) *sdiA* modestly regulates *rmf* at neutral pH. No significant changes in *rmf* expression among WT, WT + AHL, Δ*sdiA*, or Δ*sdiA* + AHL (*p* > 0.05). (**B**) *sdiA* indispensability for acid-induced *rmf* expression. At pH 2.5, *rmf* decreased by 75% in Δ*sdiA.* (**C**) Compensatory AHL signaling overcomes *rpoS* deficiency. Δ*rpoS* reduced *rmf* by 44% at pH 2.5, but AHL administration restored WT-level expression. (**D**) RMF-independent *rpoS* regulation at pH 7.0. Minimal *rpoS* change in Δ*rmf* versus WT (*p* > 0.05). (**E**) Acid-specific *rmf* induction. pH 2.5 elevated *rmf* expression 2.9-fold versus pH 7.0. (**F**) RMF sustains basal *sdiA* transcription. Δ*rmf* decreased *sdiA* by 22% at pH 7.0. (**G**) RMF enables acid-enhanced SdiA activation. Δ*rmf* reduced AHL-induced *sdiA* by 65% at pH 2.5, restricting signaling capacity to 13.8% of maximum. (**H**) RMF maintains RpoS homeostasis. Neutral pH caused 22% rpoS reduction in Δ*rmf* (*p* < 0.05). (**I**) Acid-stress synergy reveals RMF–RpoS codependence. Lethal acidity (pH 2.5) induced catastrophic 85% *rpoS* downregulation in Δ*rmf*. Data represent mean ± SD; n = 3 biological replicates. * *p* < 0.05, ** *p* < 0.01, *** *p* < 0.001, NS: not significant.

At neutral pH (7.0), *sdiA* maintained basal *rmf* expression (Δ*sdiA*: 22.5% downregulated), but AHL showed no effect (WT + AHL: 0.98 vs. WT, NS) (Figure 5A). Under acid stress (pH 2.5, 2 h), AHL and SdiA displayed synergy effects. *rmf* induction by AHL required SdiA, achieving 2.1-fold upregulation (WT + AHL: 2.10 vs. WT 1.00). Irreversible SdiA dependence: Δ*sdiA* reduced *rmf* by 75% (0.25, *p* < 0.01), and AHL failed to rescue (Δ*sdiA* + AHL: 0.63, still 37% downregulated) (Figure 5B).

Regarding RpoS functional compensation, the deletion of *rpoS* suppressed *rmf* expression by 44%, but exogenous AHL treatment fully restored *rmf* levels via SdiA-dependent signaling, achieving expression comparable to the wild-type in the Δ*rpoS* background (Figure 5C,D). Moreover, *rmf* induction exhibited strict acid dependence: pH 2.5 triggered a 2.8-fold upregulation (2.80 vs. pH 7.0 baseline) (Figure 5E).

We further investigated cross-regulatory circuitry where RMF modulates SdiA activation. Under acid stress, AHL-induced *sdiA* expression increased 4.5-fold (5.80 at pH 2.5 vs. 1.30 at pH 7.0), demonstrating pH-gated signal amplification. However, Δ*rmf* reduced basal *sdiA* expression by 65% (0.35 vs. WT 1.00), severely limiting AHL responsiveness to 13.8% of maximal activation (Figure 5F,G).

Crucially, RMF regulated RpoS abundance in a pH-dependent manner: at a neutral pH (7.0), Δ*rmf* reduced *rpoS* expression by 22% (Figure 5H), while acid stress (pH 2.5) exacerbated this defect, causing 85% downregulation in Δ*rmf* (0.15 vs. WT 1.00) (Figure 5I).

Collectively, these data support a pH-gated regulatory triad: AHL → SdiA → *rmf* → RpoS, wherein ribosomal modulation by RMF is required to maintain stress sigma factor activity during acid adaptation.

## 4. Discussion

This study establishes ribosomal hibernation as the indispensable mechanism connecting QS to acid resistance in EHEC. We demonstrate that optimized exogenous QS signaling (100 μM 3-oxo-C6-AHL for 2 h) and endogenous *yenI*-derived AHL robustly induce the ribosomal modulation factor RMF, which serves as the non-redundant executor of bacterial survival under extreme acid stress. Genetic evidence confirms that the RMF-dependent formation of 100S ribosome dimers is essential for resisting lethal acidity (pH 2.5), with *rmf* deletion causing catastrophic acid sensitivity (17-fold reduction) and abolishing QS-mediated protection against diverse stressors (including 13.7-fold increased starvation mortality). Crucially, we elucidate a pH-gated regulatory circuit where SdiA senses AHL signals to activate *rmf* specifically under acidic conditions, while RMF reciprocally sustains RpoS activity (85% *rpoS* suppression in Δ*rmf* at pH 2.5) and enhances SdiA-mediated signaling. This self-reinforcing loop represents a sophisticated adaptation mechanism that transcends conventional stress-response models.

These findings provide mechanistic insights into EHEC’s exceptional resilience, partially addressing long-standing questions regarding its ability to withstand gastric acidity despite lacking native AHL synthase. Unlike *Vibrio cholerae*—which requires high infectious doses (>10^9^ CFU) due to limited AR capacity—EHEC’s low infective dose (≈10^2^ CFU) appears to be substantially facilitated by RMF-mediated ribosomal optimization [31]. While prior research attributed acid resistance primarily to gad complexes or chaperone systems [4], our integrated data suggest that ribosomal reprogramming constitutes a major effector mechanism downstream of QS. This supports an emerging paradigm where ribosomal hibernation actively fortifies pathogens against host assaults beyond its classical role in nutrient stress adaptation.

Mechanistically, our data suggest that RMF’s role extends beyond ribosome stabilization to potentially influence global transcriptional regulation [14]. The observed severe RpoS deficiency in Δ*rmf* mutants offers an alternative perspective to conventional paradigms positioning RpoS as the apex stress regulator [12,32]. We found that *rmf* ablation substantially compromises RpoS functionality under acid stress, consistent with RMF’s putative upstream hierarchical position. This functional reorganization appears to be pathogen-specifically calibrated: whereas non-pathogenic K-12 strains primarily utilize ppGpp-dependent RMF induction [33], EHEC seems to employ acid as the dominant trigger, potentially reflecting its ecological niche adaptation. Such specialization could permit EHEC to exploit microbiome-derived AHLs via SdiA as a pre-emptive strategy for gastric challenges, showing distinctions from *Salmonella*’s direct acid-sensing strategies.

Our work further proposes a sophisticated energy allocation dynamic. While RpoS coordinates active defense by transcribing effector proteins (*gadA*/*hdeA*)—an ATP-intensive process—RMF-driven hibernation likely conserves energy by minimizing translational expenditure [5,9,15,34,35]. These potentially complementary strategies may exhibit dynamic synergy under combined QS and acid stress. During acute acid exposure (0–2 h), RMF rapidly initiates ribosome dimerization (2.9-fold *rmf* upregulation within 2 h, as measured in our assays), suggesting a prioritization of immediate energy conservation. As stress persists (>2 h), RpoS-driven effector genes become progressively predominant, potentially enabling long-term survival through active repair mechanisms. This temporal division of labor could explain why Δ*rmf* concurrently loses acid resistance and RpoS functionality: we speculate that the absence of dormancy may lead to continuous energy expenditure on futile translation, possibly exhausting resources required for RpoS activation. Ecologically, EHEC might leverage this biphasic stress response model to optimize fitness—with evidence suggesting that RMF’s rapid reaction provides immediate protection during gastric transit, while delayed RpoS activation could facilitate post-acid intestinal colonization.

Therapeutically, our findings suggest potential opportunities for disrupting ribosomal hibernation [36,37]. The significant reduction of AHL-mediated protection in Δ*rmf* strains (~99.9% impairment based on our assays) positions RMF as a candidate anti-virulence target [36]. Given that commensal-derived AHLs activate this pathway, the targeted inhibition of SdiA–RMF binding might potentially sensitize EHEC to gastric acidity with minimal impact on beneficial microbiota. The quantification of RMF’s contribution to cross-stress protection offers a provisional benchmark for evaluating novel inhibitors [19,38,39]. Furthermore, we propose that the energy-allocation paradigm could inspire innovative anti-infective strategies: simultaneously interfering with RMF-mediated energy conservation and RpoS-driven energy expenditure might synergistically affect bacterial resilience.

Several limitations warrant careful interpretation. In vitro acid challenge (pH 2.5 in LB medium) may not fully replicate gastric physiological complexities involving mucosal barriers, digestive proteases, and peristaltic dynamics. Although levels as high as 300–600 μM AHL have been reported in biofilms [40], our use of 100 μM AHL may exceed physiologically relevant concentrations in planktonic cultures. Future studies employing lower concentrations (e.g., ≤10 μM) could better elucidate AHL-mediated signaling dynamics in free-living cells. Moreover, the molecular interface between energy conservation and expenditure remains largely unresolved. For instance, how ATP/ADP ratios might influence the transition between RMF-dominated dormancy and RpoS-driven active defense requires further study [41,42,43]. Whether ribosomal hibernation spatially coordinates with virulence factor production (e.g., Shiga toxin) during infection also merits investigation. Future investigations could explore several integrated approaches: vertebrate infection models (e.g., streptomycin-treated mice) might help clarify gastric colonization defects in Δ*rmf* mutants under physiological conditions.

Collectively, our findings position ribosomal hibernation as the central adaptive mechanism bridging QS signaling to pan-stress resistance in EHEC. The SdiA–RMF–RpoS axis emerges as an integrated system coordinating environmental sensing (AHL), transcriptional regulation, and ribosomal plasticity to orchestrate dormancy-mediated survival. This work establishes RMF-driven hibernation not merely as a protective response but as the non-redundant executor of QS-enhanced resilience—explaining the catastrophic loss of acid resistance in Δ*rmf* mutants and their vulnerability to diverse host-imposed stresses. Therapeutically, targeting RMF dimerization or its crosstalk with SdiA or RpoS may offer novel anti-virulence strategies to disrupt this core survival pathway, potentially overcoming the limitations of conventional bactericidal approaches against EHEC.

## 5. Conclusions

This study establishes ribosomal hibernation—orchestrated by the Ribosomal Modulation Factor (RMF)—as the core mechanism coupling QS to acid resistance in EHEC. Experimental validation confirms that exogenous (3-oxo-C6-AHL) and endogenous AHL signaling robustly induce RMF-dependent 100S ribosome dimerization, which is indispensable for surviving lethal acidity (pH 2.5). Genetic disruption of *rmf* abolished quorum sensing-mediated protection, causing severe acid sensitivity and cross-stress vulnerability. We further elucidate a pH-gated regulatory axis (AHL → SdiA → RMF → RpoS) where RMF non-redundantly maintains RpoS functionality during acid adaptation. These results position ribosomal hibernation as a fundamental pathogenic adaptation, revealing RMF as a promising target for countering EHEC infections.

## Figures and Tables

**Table 1:** Information on ribosome-related differentially expressed genes.

Gene ID	Gene Symbol	Regulation	Fold Change	Function
EDL933_RS05+ A3:E19985	*rmf*	Up	2.9381	Ribosome Modulation Factor (RMF) promoting formation of translationally inactive 100S ribosome dimers
EDL933_RS18465	*raiA*	Up	2.0187	Ribosome-associated inactivation factors mediating translational dormancy in 70S ribosomes
EDL933_RS21670	*rpmA*	Down	−2.3985	L27, a core ribosomal protein of the 50S subunit
EDL933_RS22235	*rplO*	Up	2.2216	L15, a core ribosomal protein of the 50S subunit
EDL933_RS21870	*rplM*	Down	−2.6348	L13, a core ribosomal protein of the 50S subunit
EDL933_RS16425	*rplY*	Down	−2.5399	L25, a core ribosomal protein of the 50S subunit
EDL933_RS22375	*rpsL*	Down	−2.6338	S12, a core ribosomal protein of the 30S subunit
EDL933_RS21050	*rpsU*	Down	−2.6047	S21, a core ribosomal protein of the 30S subunit
EDL933_RS00125	*rpsT*	Down	−2.4991	S20, a core ribosomal protein of the 30S subunit
EDL933_RS22335	*rpsJ*	Down	−1.54	S10, a core ribosomal protein of the 30S subunit
EDL933_RS21675	*rplU*	Down	−2.1187	L21, a core ribosomal protein of the 50S subunit
EDL933_RS21595	*rimP*	Down	−1.9906	Ribosome maturation enzymes catalyzing structural remodeling of 30S ribosomal subunits
EDL933_RS21645	*yhbY*	Down	−1.7577	Ribosome dimerization factors binding and stabilizing mature 50S ribosomal subunits
EDL933_RS02525	*tig*	Down	−3.0643	Ribosome-associated chaperones coordinating nascent chain folding, protein translocation, and ribosome maturation

## Data Availability

The original contributions presented in this study are included in the article/Supplementary Material. Further inquiries can be directed to the corresponding authors.

## Supplementary Materials

The following supporting information can be downloaded at: https://www.mdpi.com/article/10.3390/microorganisms13081730/s1, Table S1: Strains and plasmids used in this study; Table S2: Primers used in this study; Figure S1: Molecular Verification of RMF Deletion Mutants in EHEC; Figure S2: RMF Deficiency Exacerbates Susceptibility to Oxidative and Environmental Stresses in EHEC.
