# Supplementary material for: Ribosomal Hibernation Factor Links Quorum-Sensing to Acid Resistance in EHEC
Source: Microorganisms. 2025 Jul 24;13(8):1730. doi: 10.3390/microorganisms13081730 (PMC12388267; doi:10.3390/microorganisms13081730)
Supplement: Supplementary file 1 [file microorganisms-13-01730-s001.zip › microorganisms-3735879-supplementary.pdf]

Tables

Table S1. Strains and plasmids used in this study.

Strains and plasmids	Details	Selection of Antibiotics Used	Reference
**Strains**			
EDL933	Wild-type (O157:H7: *stx*1$^+$ *stx*2$^+$ *eae*$^+$ *hly*$^+$ *escV*$^+$)	-	Preserved in our laboratory
EDL933/p*yenI*	EDL933 carrying p*yenI*	Amp$^+$	Constructed in this study
EDL933 Δ*rmf*	*rmf* knockout strain	-	Constructed in this study
EDL933 Δ*rmf*/ p*rmf*	*rmf* knockout strain complemented with p*rmf*	Amp$^+$	Constructed in this study
EDL933Δ*rmf*/p*yenI*	EDL933 *rmf* knockout strain carrying p*yenI*	Amp$^+$	Constructed in this study
EDL933 Δ*sdiA*	*sdiA* knockout strain	-	Constructed previously and preserved in our laboratory
EDL933 Δ*rpoS*	*rpoS* knockout strain	-	Constructed previously and preserved in our laboratory
C. violaceum CV026	AHL biosensor	-	Preserved in our laboratory
**Plasmids**			
pBR322	Expression vector, Amp^r	Amp$^+$	Takara Ltd.
p*yenI*	pBR322 carrying LuxI ORF	Amp$^+$	Constructed previously and preserved in our laboratory
pKD3	Cm^r; Cm cassette template	Cm$^+$	Preserved in our laboratory
pKD46	Amp^r, λRed recombinase	Amp$^+$	Preserved in our laboratory
pCP20	Amp^r, Cm^r; Flp recombinase	Amp$^+$ Cm$^+$	Preserved in our laboratory

Table S2 Primers used in this study

Primers	Sequencing (5'-3')	Purpose
gapA-RT-F	GGCAAACTGACTGGTATGGC	RT-qPCR
gapA-RT-R	CATCTTCGGTGTAGCCCAGA	RT-qPCR
raiA-RT-F	AAGGGTTTGTTGCTGACGC	RT-qPCR
raiA-RT-R	GTTGGCGTCTTTCACCGATG	RT-qPCR
rmf-RT-F	CTGGAACGGGCACATCAAC	RT-qPCR
rmf-RT-R	TTACTACCCTGTCCGCCATG	RT-qPCR
rpmA-RT-F	CGAATTTCACTTTACCGTCTGC	RT-qPCR
rpmA-RT-R	TCGCGATTCAGAAGCTAAACG	RT-qPCR
rplO-RT-F	GGTCTACTACACCGCCTTCT	RT-qPCR
rplO-RT-R	GGTTCTGGCCTCGGTAAAAC	RT-qPCR
rplM-RT-F	ACATGCCTTTAACCGCGATT	RT-qPCR
rplM-RT-R	AACGCTGACAAAGTTGCTGT	RT-qPCR
rplY-RT-F	ACAAGTTCCCGGCAATCATC	RT-qPCR
rplY-RT-R	GCTGTACGTCCTGAGCTTTA	RT-qPCR
rplU-RT-F	TTAACTTTCTCGCCACGACC	RT-qPCR
rplU-RT-R	CCTGGAAAAGCTGGACATCG	RT-qPCR
rpsL-RT-F	TGCTTACGGTCTTTAACGCC	RT-qPCR
rpsL-RT-R	AGTGACTTCCTACATCGGTGG	RT-qPCR
rpsT-RT-F	CAGACCTTTAGCAGCCTGAC	RT-qPCR
rpsT-RT-R	TCAGTCTGAAAAGGCTCGTAAG	RT-qPCR
rpsJ-RT-F	CGGGGAGATCAGAACAGTGA	RT-qPCR
rpsJ-RT-R	AGAATCCGTATCCGCCTGAA	RT-qPCR
rimP-RT-F	CCTGCCATTTACGACGGTTT	RT-qPCR
rimP-RT-R	GAAGATCCCATCACCGTTGC	RT-qPCR
yhbY-RT-F	GAAGATCCCATCACCGTTGC	RT-qPCR
yhbY-RT-R	GCGTTTTACCGATGACCTGT	RT-qPCR
tig-RT-F	CAGAAGACCGCGTAACCATC	RT-qPCR
tig-RT-R	AACTCTTCGCCAGCTTTGTG	RT-qPCR
rmf -P1	ATGAAGAGACAAAAACGAGAT	Cloning

rmf -P2	TCAGGCCATTACTACCCT	Cloning
rmf-knockout-P3	ATGAAGAGACAAAAACGAGATCGCCTGGAACGGGC<br>ACATCAACGTGGTTATGTGTAGGCTGGAGCTGCTTCG	Knockout
rmf-knockout-P4	GCGTCTGATAGGGACACATTTCTTTGAGCGTCCGGC<br>GATGCCGGCCTGACATATGAATATCCTCCTTAG	Knockout
rmf-complemented-P5	CGC GGATCCATGAAGAGACAAAAACGAGATC	Cloning
rmf-complemented-P6	CAG GTCGACTCAGGCCACTACCCTGT	Cloning
rpoS-RT-F	ACATCGTAAAGGAGCTGAACGT	RT-qPCR
rpoS-RT-R	ATCTCTTCCGCACTTGGTTCAT	RT-qPCR
sdiA-RT-F	CGCGCGAAATACCCATTCTTAG	RT-qPCR
sdiA-RT-R	GCTGAAATTCATCTCTGGCGTC	RT-qPCR
gadA-RT-F	ATGAGTTCATCTGTACCGGTCG	RT-qPCR
gadA-RT-R	TCAGAGAGGTCATACAGGGTGT	RT-qPCR
gadC-RT-F	CGGTTTGTCTATTGCGATGGTC	RT-qPCR
gadC-RT-R	GGGACATCAGAACGGTAAAGGT	RT-qPCR
gadW-RT-F	TTGCGTTTTGTTCACCGGATAC	RT-qPCR
gadW-RT-R	CTAAATTGCGTGGTAGCTGACG	RT-qPCR
gadE-RT-F	TCGACGCTCAATATTTCGCAAC	RT-qPCR
gadE-RT-R	AAGATGTGATACCCAGGGTGAC	RT-qPCR
hdeA-RT-F	TGGACCTGTGAAGATTTCCTGG	RT-qPCR
hdeA-RT-R	TCTTTGTTGTTCAGCGCTTCAG	RT-qPCR
hdeB-RT-F	TCCGCTAAAGATATGACCTGCC	RT-qPCR
hdeB-RT-R	TGTTTCTTCATGCAGCATCCAC	RT-qPCR
adiA-RT-F	AATGGGGGACTCTGGTTAACAC	RT-qPCR
adiA-RT-R	TTGTTCAACAAGTTCCGGCATC	RT-qPCR

Figures

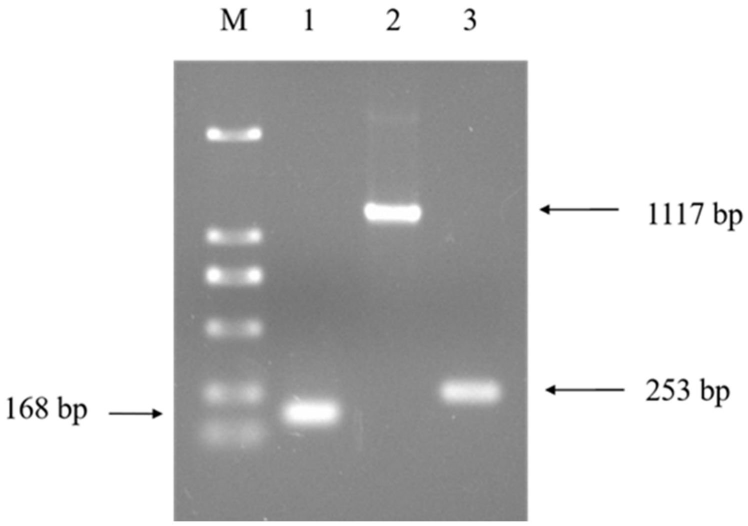

Figure S1. Molecular verification of RMF deletion mutants in EHEC.

PCR-based genotyping of Δ*rmf* derivatives. The 1117 bp amplicon in lane 2 confirms retention of the chloramphenicol resistance cassette (cat) in primary recombinant EDL933 Δ*rmf*::cat. Successful excision of cat yields the 253 bp product in EDL933 Δ*rmf* (lane 3), contrasting with wild-type amplification (lane 1). Samples resolved on 1.2% agarose gel with Trans 2K DNA Marker reference (lane M) (template preparation: boiling lysis; primers: P1/P2).

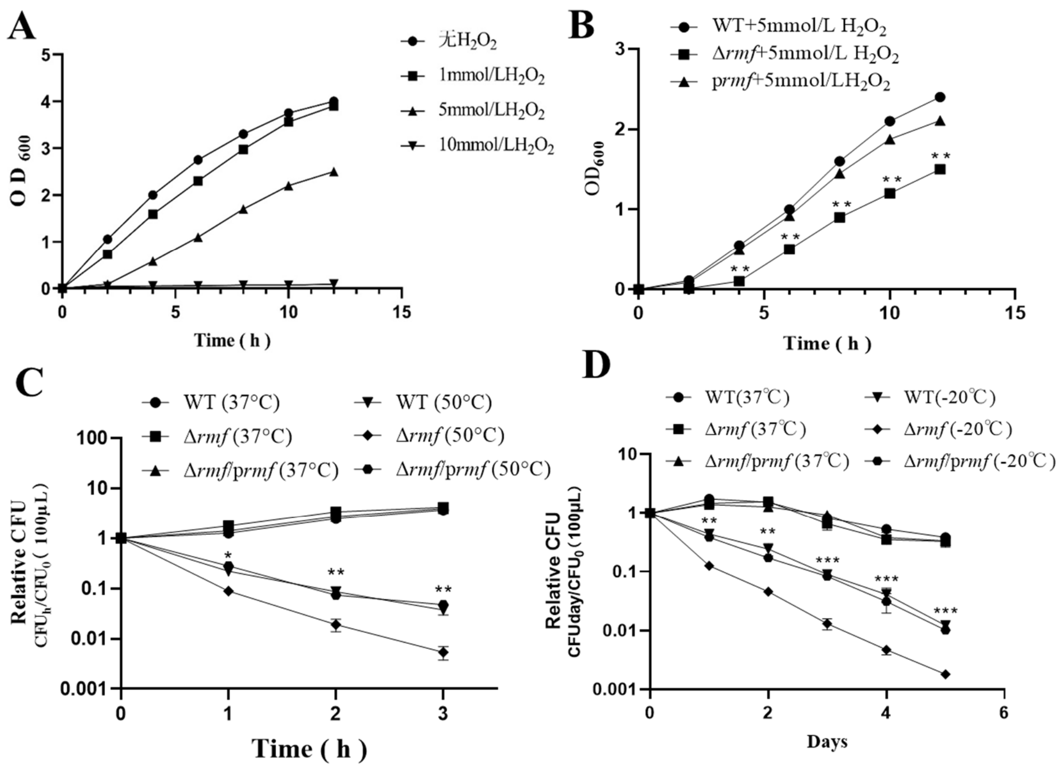

Figure S2. RMF deficiency exacerbates susceptibility to oxidative and environmental stresses in EHEC.

Stationary-phase cultures were normalized to OD_{600} = 1.0 in pre-warmed LB (pH 7.4). Stress resilience was evaluated under four environmentally relevant conditions. Oxidative stress: Mid-exponential phase cultures (OD_{600} = 0.6 ± 0.05) were exposed to 5μM H_2O_2 in LB broth. Cold stress: Bacterial suspensions normalized to OD_{600} = 1.0 were held at -20°C. Thermal stress: Cultures were incubated in a precision-controlled 50°C water bath. At specified intervals (0, 1, 2, and 3 h), samples were immediately diluted 10-fold in ice-cold PBS, subjected to serial dilutions, and plated on LB agar. Viable colonies were enumerated after 12 h incubation at 37°C. Survival rates were calculated as CFU at time h (CFU_h) normalized to t=0 values (CFU_0). (**A**) H_2O_2 dose-response impairs bacterial growth. Untreated wild-type EHEC EDL933 displays logarithmic growth over 12 h. Subinhibitory H_2O_2 attenuates growth, whereas 10 mmol/L abolishes replication. The 5 mmol/L concentration was chosen for subsequent oxidative stress assays. (**B**) RMF is essential for oxidative stress adaptation. When challenged with 5 mmol/L H_2O_2, the Δ*rmf* mutant exhibits significantly impaired growth versus the wild-type, evidenced by a 50% reduction in OD_{600} at 9 h. Partial phenotypic restoration occurs in the complemented strain Δ*rmf/*p*rmf.* (**C**) Thermosensitivity of Δ*rmf* mutant. At 50°C, wild-type viability decreases to 3.75% after 3 h, while Δ*rmf* survival collapses to 0.53%—representing a 7.0-fold reduction relative to the wild-type. Genetic complementation partially rescues heat susceptibility. (**D**) Cryosensitivity of Δ*rmf* mutant. During -20°C exposure, the wild-type maintains 1.21% survival by day 5, contrasting with 0.18% in Δ*rmf* (6.8-fold decrease). The complemented strain shows intermediate tolerance.
